# One-Year Mortality in Older Patients with Cancer: Development and External Validation of an MNA-Based Prognostic Score

**DOI:** 10.1371/journal.pone.0148523

**Published:** 2016-02-09

**Authors:** Isabelle Bourdel-Marchasson, Abou Diallo, Carine Bellera, Christelle Blanc-Bisson, Jessica Durrieu, Christine Germain, Simone Mathoulin-Pélissier, Pierre Soubeyran, Muriel Rainfray, Mariane Fonck, Adelaïde Doussau

**Affiliations:** 1 Clinical Gerontology Department, CHU Bordeaux, Bordeaux, France; 2 RMSB, UMR 5536, CNRS, Bordeaux, France; 3 RMSB, UMR 5536, Bordeaux University, Bordeaux, France; 4 Clinical Epidemiology Unit, CHU Bordeaux, Bordeaux, France; 5 CIC-14.01, INSERM, Bordeaux, France; 6 Clinical Research and Clinical Epidemiology Unit, Institut Bergonié, Bordeaux, France; 7 Medical Oncology Department, Institut Bergonié, Bordeaux, France; 8 Bordeaux University, Bordeaux, France; INRS, CANADA

## Abstract

**Purpose:**

The MNA (Mini Nutritional Assessment) is known as a prognosis factor in older population. We analyzed the prognostic value for one-year mortality of MNA items in older patients with cancer treated with chemotherapy as the basis of a simplified prognostic score.

**Methods:**

The prospective derivation cohort included 606 patients older than 70 years with an indication of chemotherapy for cancers. The endpoint to predict was one-year mortality. The 18 items of the Full MNA, age, gender, weight loss, cancer origin, TNM, performance status and lymphocyte count were considered to construct the prognostic model. MNA items were analyzed with a backward step-by-step multivariate logistic regression and other items were added in a forward step-by-step regression. External validation was performed on an independent cohort of 229 patients.

**Results:**

At one year 266 deaths had occurred. Decreased dietary intake (p = 0.0002), decreased protein-rich food intake (p = 0.025), 3 or more prescribed drugs (p = 0.023), calf circumference <31cm (p = 0.0002), tumor origin (p<0.0001), metastatic status (p = 0.0007) and lymphocyte count <1500/mm^3^ (0.029) were found to be associated with 1-year mortality in the final model and were used to construct a prognostic score. The area under curve (AUC) of the score was 0.793, which was higher than the Full MNA AUC (0.706). The AUC of the score in validation cohort (229 subjects, 137 deaths) was 0.698.

**Conclusion:**

Key predictors of one-year mortality included cancer cachexia clinical features, comorbidities, the origin and the advanced status of the tumor. The prognostic value of this model combining a subset of MNA items and cancer related items was better than the full MNA, thus providing a simple score to predict 1-year mortality in older patients with an indication of chemotherapy.

## Introduction

The question of prognosis is crucial before starting a treatment for cancer. The Comprehensive Geriatric Assessment (CGA) can offer a prognostic evaluation in older patients with cancer [1–4]. We have previously reviewed the central role of nutritional parameters in the CGA and their capacity to predict mortality in older people [5]. It is recommended by French Speaking Society of Clinical Nutrition and Metabolism (SFNEP) to screen malnutrition in patients during their treatment for cancer [6]. The suggested tools are weight loss search, dietary intake analogue scale, and multidimensional screening tools such as the full MNA (Mini Nutritional Assessment) in older, and the scored Patient-Generated Subjective Global Assessment (PG-SGA) in adult. Undernourished or at-risk-for-malnutrition subjects with cancer according to the MNA (Mini Nutritional Assessment) [7] are at increased risk for one-year mortality in multi-type cancer cohorts with indications for chemotherapy [2, 3]. MNA, toxicity of the treatment regimen, MMSe (Mini Mental State examination) and performance status are included in a score for prediction of non-hematologic toxicities in patients receiving chemotherapy [1]. The prognostic value of the MNA in older patients is not limited to cancer. In an older community-living population in Taiwan, an adapted form of the MNA predicted mortality: the rate was the highest for malnourished subjects and was intermediate in subjects at risk for malnutrition [8]. A similar prognostic value of the MNA was found in hospitalized patients [9]. The MNA is a multi-component scale including nutritional data such as food intake data and anthropometry and health-related quality of life data such as functional dependency, mental health, diseases, prescribed drugs and subjective health assessment in the field of nutrition and in general. The G8 tool, which was proposed as a screening tool for vulnerability, consists of seven items from the original 18-item MNA (appetite changes, weight loss, mobility, neuropsychological problems, body mass index, medication, and self-rated health) and patient’s age. G8 was found predictive of abnormal CGA [10]. The full-length scale thus includes most of the known prognostic factors in cancer [11–15] with the exception of disease-related prognostic factors.

The objective of this study was to evaluate the prognostic value for one-year mortality of items included in the Full MNA or in the short form of the MNA in patients with cancer. The secondary objective was to construct and validate a composite score predicting one-year mortality, based on a model including MNA items and the other known prognostic factors discussed above.

## Methods

### Patients

The derivation cohort was the screening population of a multicenter randomized clinical trial testing the effect of dietary advice on mortality within the group of older patients with cancers or lymphoma at risk for malnutrition according to the MNA during chemotherapy, conducted between 2007 and 2012 [16]. During the screening procedure, Full MNA and a short description of cancer type, metastatic status (or prognostic index for lymphoma), weight changes, ECOG (Performance Status Eastern Cooperative Oncology Group) status and biological data were collected. The institutional Review Board of South-West France and Overseas French departments, France, approved the study protocol. The patients were proposed a written informed consent to participate in the RCT only if they were at risk for malnutrition according to full MNA. The institutional review board has approved the follow up for mortality of all patients who were screened.

The institutional Review Board of South-West France and Overseas French departments, France, approved the follow-up of all screened subjects for 2-year mortality

All patients older than 70 with a planned first to third line chemotherapy, in 11 recruiting centers were screened using the Full MNA to participate in a randomized study testing the effects of dietary advices. This was the first line chemotherapy for 80.0%, the second for 14.9% and the third for 5.2%. The main outcome of this trial was one-year mortality. No difference in mortality was found between the two randomized groups (usual care versus usual care +dietary advice). Cancers sites were the colon, stomach, pancreas and biliary ducts, ovary, prostate, bladder, breast and non-small-cell lung and lymphoma. Further details and results of the randomized trial are described elsewhere [16].

An independent observational cohort was used to validate the model in order to evaluate its generalizability. The 364 subjects older than 70 y, had an indication of first-line chemotherapy for a cancer from lung, colon, stomach, pancreas, ovary, bladder, prostate or from unknown origin, and for lymphoma and their characteristics have been published elsewhere [17]. No breast or biliary duct cancer was included in this cohort.

The present study included all subjects of those two cohorts except those with a lymphoma or those without a MNA score.

### Statistical Considerations

One-year mortality was the primary endpoint and the inclusion day was the origin of the follow-up. The multivariate strategy was aimed at prioritizing the selection of the MNA variables over clinical variables. Indeed the primary objective was to reduce the number of considered MNA items, and the secondary objective was to improve the capacity of a relevant subset of MNA items to predict one-year mortality, using some additional clinical factors easily accessible at the bedside of the patient.

Potential factors for this prognostic model were retrieved from baseline assessment and included MNA and other clinical data. All of the 18 items in the MNA were explored in the modelling procedure. Items were categorized as presented in the MNA questionnaire (see forms on http://www.mna-elderly.com/mna_forms.html). The clinical factors included age, weight loss, cancer origin, TNM staging, PS-ECOG, and lymphocytes count. Age was categorized into 3 classes: <75y, 75-79y and 80y and older [18]. Weight loss was considered in four classes: no weight loss (reference), weight loss < 5%, weight loss from 5% to < 10% and 10% or greater amount of weight loss. T stage was categorized into 5 classes, as undetermined stages were considered as a separate category, based on the hypothesis that in the elderly, the inability to evaluate TNM stage might be a marker of severity. Similarly, undetermined N and M stage categories were considered as separate categories. PS-ECOG was categorized into 4 classes from patients with no activity restriction to patients bedridden more than 50% of the time. Lymphocyte count was categorized into 2 classes: < 1500/mm3 and 1500/mm3 or more. The prognostic values of different forms of MNA were studied for a one-point increase in each score: the Full MNA (18 items, maximal score 30), the MNA (6 items maximal score 14) including the first 5 items of the Full MNA and with two different 6^th^ items, BMI or calf circumference (CC) [19].

In the derivative cohort, we used univariate and multivariate logistic models to estimate odds ratio (OR) and 95% confidence interval (95% CI). All variables with p-values <0.20 in the univariate analyses were eligible for the multivariate model. The first step of the multivariate analysis included only the items of the MNA. A step-by-step backward strategy was performed to select within the MNA items those independently associated with one-year mortality with a p<0.05. The second step included the significant items of the MNA independently associated with one-year mortality. Clinical variables described above among well-known prognosis factors selected in univariate analysis were then introduced following a step-by-step forward strategy. The threshold of 0.05 for statistical significance was used to maintain the variable in the model. Weight loss item or functional items of the MNA and from clinical data were not considered in the model altogether therefore avoiding collinearity. A prognostic score was constructed with the variables of the final model by multiplying the regression parameters of the logistic model by 10. The 1-year probability of death can be estimated using the inverse logit function [20]. Finally, an external validation was performed on the independent cohort of patients with cancer [3].

For both the derivative and validation cohorts, discrimination was evaluated through the area under curve (AUC) of the score and ROC curves, and calibration was represented through calibration plots. Calibration plots make it possible to compare observed and predicted event rates; discrimination can be used to quantify the score’s ability to distinguish between patients who do or do not experience the event [17]. AUC were compared with a non-parametric test [21].

## Results

**Fig 1:**
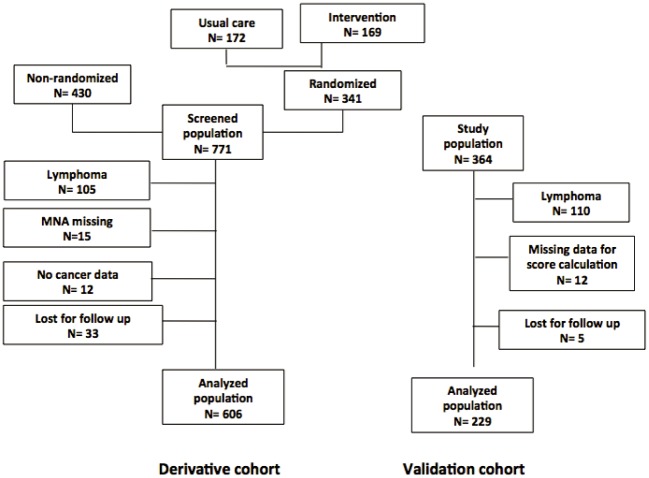
Flow chart of patients included in derivative cohort based on screening for participation in randomized controlled study and in validation cohort.

The initial population screened to participate in the randomized clinical trial included 771 patients (Fig 1). After exclusion of patients with lymphoma (105), those with no indication of the origin of cancer (12), those with no MNA score (15) and those lost to follow-up (N = 33), the study population included 606 patients. Baseline characteristics of the study population are presented in Table 1. According to the MNA score, 78 (12.9%) patients were malnourished (MNA <17), 317 (52.3%) were at risk for malnutrition (MNA ranging from 17 to 24) and 211 (34.8%) were well nourished (MNA> = 24).

**Table 1:** Baseline Characteristics of Study populations and Univariate Analysis of One-year Mortality Prediction in Derivative Cohort.

	Derivative cohort				Validation cohort
	N = 606 (%)	One-year mortality N (%)	OR [95% CI]	*p*-value †	n = 229 (%)
**Age**				**0.03**	
Age<75 y old	196 (32.3)	72(36.7)	1		84(36.7)
75< = age< 80 y old	227 (37.5)	103(45.4)	1.43 [0.97; 2.11]		76(33.2)
Age> = 80 y old	183 (30.2)	91(49.7)	1.70 [1.13; 2.57]		69(30.1)
**Gender**				**<0.05**	
Male	319 (52.6)	152(47.6)	1.38 [1.00; 1.91]		143(62.4)
**Weight loss**				**0.006**	
None	88 (14.5)	28(31.8)	1		
<5%	77 (12.7)	31(40.3)	1.42 [0.76; 2.74]		
> = 5%and <10%	114 (18.8)	57(50.0)	2.14 [1.20; 3.82]		
> = 10%	163 (27.1)	86(52.8)	2.35 [1.43: 3.87]		
Missing	164 (26.9)	64(39.0)	2.39 [1.39; 4.12]		
**T Stage**				**0.10**	
1	14 (2.3)	1(7.1)	1		7 (3.1)
2	71 (11.7)	27(38.0)	7.;98 [0.99; 64.45]		25 (10.9)
3	148 (24.4)	65(43.9)	10.18 [1.30; 79.83]		69 (30.1)
4	133 (21.9)	63(47.4)	11.70 [1.49; 91.98]		37 (16.2)
Missing and undetermined	240 (39.6)	110(45.8)	11.00 [1.42; 85.40]		91 (39.7)
**N stage**				**0.002**	
No	55 (9.1)	12 (21.8)	1		30 (13.1)
Yes	333 (55.0)	147 (44.1)	2.83 [1.44; 5.56]		72(31.4)
Missing and undetermined	218 (36.0)	107 (49.1)	3.45 [1.73; 6.91]		127(55.5)
**Metastasis (M)**				**<0.0001**	
No	197 (32.5)	58(29.4	1		83 (36.2)
Yes	368 (60.7)	190(51.6)	2.56 [1.77; 3.70]		
Missing and undetermined	41 (6.8)	18(43.9)	1.88 [0.94; 3.73]		20 (8.7)
**Cancer origin**				**<0.0001**	
Non-small cell lung	68 (11.2)	46(67.6)	5.097 [2.77; 9.37]		35 (15.3)
Colon	165 (27.2)	48(29.1)	1		87 (38.0)
Stomach	56 (9.2)	28(50.0)	2.44 [1.31; 4.54]		33 (14.4)
Ovary	52 (8.6)	19(36.5)	1.40 [0.73; 2.71]		13 (5.7)
Pancreas	76 (12.5)	51(67.1)	4.97 [2.77; 8.923]		21 (9.2)
Cholangiocarcinoma	15 (2.5)	8(53.3)	2.79 [0.96; 8.11]		0
Unknown	3 (0.5)	1(33.3)	1.22 [0.11; 13.76]		4 (1.7)
Prostate	50 (8.3)	21(42.0)	1.76 [0.92; 3.40]		19 (8.3)
Breast	72 (11.9)	20(27.8)	0.94 [0.51; 1.731]		0
Bladder	49 (8.1)	24(49.0)	2.34 [1.22; 4.50]		17 (7.4)
**ECOG**				**<0.0001**	
Missing	207 (34.2)	88 (42.5)			10 (4.4)
0- Fully active, able to carry on all pre-disease performance without restriction	133 (21.9)	38 (28.6)	1		45 (19.7)
1- Restricted in physically strenuous activity but ambulatory and able to carry out work of a light or sedentary nature, e.g., light house work, office work	174 (28.7)	79 (45.4)	2.08[1.29; 3.36]		
2- Ambulatory and capable of all self-care but unable to carry out any work activities. Up and about more than 50% of waking hours	70 (11.6)	45 (64.3)	4.50 [2.43; 8.34]		38 (16.6)
3-Dependent	22 (3.6)	16 (72.7)	6.67 [2.43; 18.32]		10 (4.4)
**Lymphocytes**				**<0.0001**	
Missing	41 (6.8)	15 (36.6)			0
<1500/mm3	313 (51.7)	165 (52.7)	2.15 [1.53; 3.03]		126 (55.0)
> = 1500/mm3	252 (41.6)	86 (34.1)	1		103 (45.0)

^†^ Wald Chi2 test

The scores for the 3 MNA forms and scores for all 18 items of the MNA are presented in Table 2. There were very few patients bed- or chair-bound, severely depressed or demented, with skin ulcers, who ate less than 2 meals per day, who had no fruit and vegetable intake or who drank fewer than 3 cups of fluid per day, who needed assistance for feeding and who had a low mid-arm circumference.

**Table 2:** Baseline Full MNA Items in the Derivative Population and Univariate Analysis of One-year Mortality Prediction.

	N = 606 (%)	One-year death N (%)	OR [95% CI]	*p*-value †	AUC
**Food intake decrease over the last 3 months**				**<0.0001**	
Severe decrease	81 (13.4)	57 (70.4)	6.07 [3.53; 10.45]		
Moderated decrease	244 (40.3)	130 (53.3)	2.92 [2.03; 4.19]		
No decrease	281 (46.4)	79 (28.1)	1		
**Weight loss during the last 3 months**				**0.0005**	
Weight loss > 3 kg	282 (46.5)	144 (51.1)	2.15 [1.47; 3.15]		
Does not know	22 (3.6)	13 (59.1)	2.98 [1.21; 7.34]		
Weight loss between 1 and 3 kg	109 (18.0)	46 (42.2)	1.51 [0.93; 2.45]		
No weight loss	193 (31.8)	63 (32.6)	1		
**Mobility**				**0.0009**	
Bed- or chair-bound	25 (4.1)	17 (68.0)	3.17 [1.34; 7.49]		
Able to get out of bed / chair but does not go out	100 (16.5)	56 (56.0)	1.90 [1.230; 2.93]		
Goes out	481 (79.4)	193 (40.1)	1		
**Psychological stress or acute disease in the last 3 months**					
Yes	606 (100)	266 (43.9)	1		
**Neuropsychological problems**				**0.0003**	
Severe dementia or depression	25 (4.1)	13 (52.0)	1.73 [0.77; 3.88]		
Mild dementia or depression	158 (26.1)	90 (57.0)	2.11 [1.46; 3.06]		
No psychological problems	423 (69.8)	163 (38.5)	1		
**BMI, kg/m**^**2**^				**0.0002**	
**BMI** <19	43 (7.1)	25 (58.1)	2.41 [1.26; 4.58]		
19< = **BMI** <21	78 (12.9)	44 (56.4)	2.24 [1.36; 3.68]		
21< = **BMI** <23	127 (21.0)	66 (52.0)	1.87 [1.24; 2.82]		
**BMI** > = 23	358 (59.1)	131 (36.6)	1		
**Living independently at home**				**0.0004**	
No	86 (14.2)	53 (61.6)	2.31 [1.45; 3.70]		
Yes	520 (85.8)	213 (41.0)	1		
**Takes more than 3 prescription drugs per day**				**0.0009**	
Yes	379 (62.5)	186 (49.1)	1.77 [1.26; 2.48]		
No	227 (37.5)	80 (35.2)	1		
**Pressure sores or skin ulcers**				**0.90**	
Yes	40 (6.6)	18 (45.0)	1.049 [0.55; 2.00]		
No	566 (93.4)	248 (43.8)	1		
**Number of daily full meals**				**0.0001**	
1 meal	21 (3.5)	16 (76.2)	4.69 [1.69; 13.01]		
2 meals	57 (9.4)	36 (63.2)	2.51 [1.43; 4.43]		
3 meals	528 (87.1)	214 (40.5)	1		
**Protein-rich food intake**				**<0.0001**	
1 low	43 (7.1)	33 (76.7)	5.73 [2.75; 11.94]		
2 intermediate	139 (22.9)	78 (56.1)	2.22 [1.50; 3.27]		
3 high	424 (70.0)	155 (36.6)	1		
**Two or more servings of fruit or vegetables per day**				**0.009**	
No	54 (8.9)	33 (61.1)	2.15 [1.21; 3.81]		
Yes	552 (91.1)	233 (42.2)	1		
**Fluid intake**				**0.10**	
Fewer than 3 cups	14 (2.3)	9 (64.3)	2.47 [0.81; 7.47]		
3 to 5 cups	99 (16.3)	49 (49.5)	1.34 [0.87; 2.07]		
More than 5 cups	493 (81.4)	208 (42.2)	1		
**Mode of feeding**				**0.06**	
Unable to eat without assistance	9 (1.5)	6 (66.7)	2.70 [0.67; 10.9]		
Self-fed with some difficulty	33 (5.4)	20 (60.6)	2.08 [1.01; 4.26]		
Self-fed without any problem	564 (93.1)	240 (42.6)	1		
**Self-view of nutritional status**				**<0.0001**	
Views self as being malnourished	21 (3.5)	13 (61.9)	2.78 [1.13; 6.86]		
Uncertain of nutritional state	159 (26.2)	96 (60.4)	2.61 [1.87; 3.79]		
No nutritional problem	426 (70.3)	157 (36.9)	1		
**Self-view of health status in comparison with other people of the same age**				**<0.0001**	
Not as good	149 (24.6)	95 (63.8)	4.99 [3.02; 8.25]		
Does not know	83 (13.7)	38 (45.8)	2.40 [1.35; 4.24]		
As good	232 (38.3)	96 (41.4)	2.00 [1.27; 3.16]		
Better	142 (23.4)	37 (26.1)	1		
**Mid-arm circumference (MAC), cm**				**0.10**	
MAC<21	29 (4.8)	18 (62.1)	2.21 [1.02; 4.76]		
21< = MAC< = 22	32 (5.3)	16 (50.0)	1.35 [0.66; 2.75]		
MAC>22	545 (89.9)	232 (42.6)	1		
**Calf circumference (CC) cm**				**<0.0001**	
CC<31	85 (14.0)	56 (65.9)	2.86 [1.77; 4.63]		
CC> = 31	521 (86.0)	210 (40.3)	1		
**MNA score with BMI in 6**^**th**^ **question**			0.78 [0.73; 0.84]	**<0.0001**	**0.671**
(for 1-point increase)					
**MNA score with CC in 6**^**th**^ **question**			0.76 [0.71; 0.82]	**<0.0001**	**0.679**
(for 1-point increase)					
**Full MNA score**			0.82 [0.79; 0.86]	**<0.0001**	**0.706**
(for 1-point increase)					

^†^ Wald Chi2 test

### One-year mortality

At one year, 266 patients were deceased. For almost all of them, the declared cause of death was the cancer itself (244 patients, 91.7%). Other causes of death were cancer treatment toxicities associated with cancer in 5 patients, a cancer other than the initial one in 3 patients and an intercurrent event or with no information on cause in only 7 (2.6%) patients. The 1-year mortality incidence was 70.5% in malnourished patients, 48.9% in those at risk for malnutrition, and 26.5% in those considered as well nourished. The three MNA forms scores were strongly associated with one-year mortality (Table 2), and AUC ranged from 0.671 to 0.706. With the exception of T staging, all candidate predictors in known baseline characteristics of patients were associated with mortality in univariate analysis (Table 1).

### Development of a prognostic score

**Fig 2:**
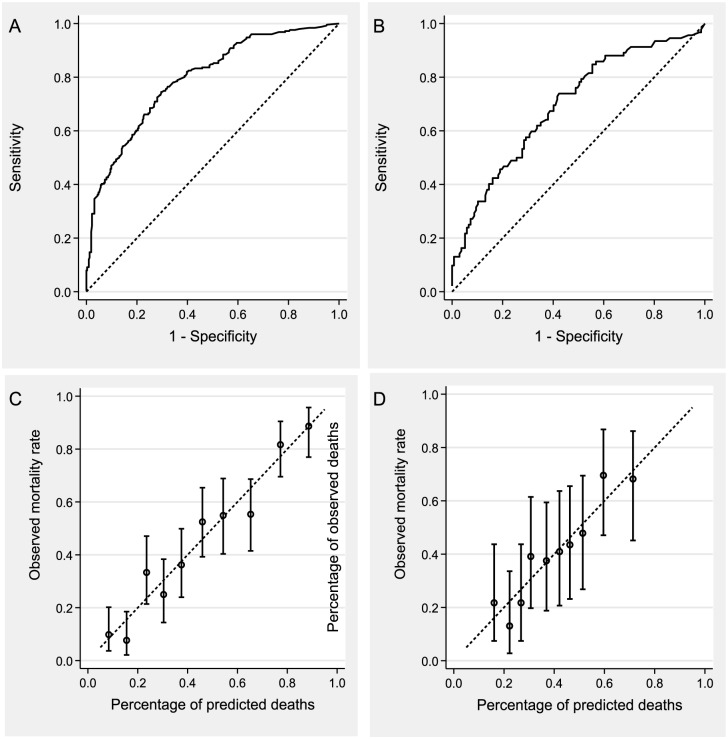
ROC curves of predictive score among derivative population (A) and validation population (B) and respective calibration plots (C and D). Vertical bars correspond to 95%.

The multivariate model showed that 5 items of MNA were independently associated with one-year mortality: decreased food intake, taking 3 or more prescribed drugs, low protein-rich food intake (nutritional supplement not included), self-view of health status assessed as similar or worse than other persons of the same age and calf circumference lower than 31 cm (Table 3). After addition of the baseline characteristics of the patients, the self-rated health status from the prognostic variables was eliminated, whereas origin of cancer, existence of metastasis or missing and undetermined and lymphocyte count lower than 1500/mm^3^ were added (Table 3). The AUC of the final model was 0.793. This model had a better prognostic discrimination value than MNA scores: the AUC of the Full MNA was lower than that of the final model (0.712 in 565 patients of the final model, as compared to 0.793, p<0.0001) (Fig 2) and was thus used to construct a prognostic score.

**Table 3:** Multivariate Models of One-year Mortality Prediction (Derivative Cohort) and Corresponding Sub-score Values confidence interval of observed mortality rate.

	MNA item Model, N = 606, AUC 0.729		Final Model, N = 565, AUC 0.793			
	OR [95% CI]	p-value	OR [95% CI]	p-value	beta	Sub-score value
**Food intake over the last 3 months**		**<0.0001**		**0.0002**		
Severe decrease	3.14 [1.68; 5.89]		3.82 [1.87; 7.79]		**1.34**	13.4
Moderate decrease	2.30 [1.56; 3.39]		2.03 [1.31; 3.14]		**0.71**	7.1
No decrease	1		1			0.0
**Takes more than 3 prescription drugs per day**		**0.01**		**0.02**		
Yes	1.61 [1.11; 2.33]		1.62 [1.07; 2.44]		**0.48**	4.8
No	1		1			0.0
**Protein-rich food intake**		**0.03**		**0.03**		
Low	2.72 [1.17; 6.19]		2.71 [1.07; 6.88]		**1.00**	10.0
Intermediate	1.47 [0.96; 2.27]		1.69 [1.04; 2.73]		**0.52**	5.2
High	1		1			0.0
**Self-view of health status in comparison with other people of the same age**		**0.005**				
Not as good	2.55 [1.47; 4.43]					
Does not know	1.29 [0.69; 2.41]					
As good	1.76 [1.09; 2.84]					
Better	1					
**Calf circumference (CC), cm**		**0.002**		**0.0002**		
CC<31	2.27 [1.36; 3.80]		3.08 [1.69; 5.61]		**1.13**	11.3
CC> = 31	1		**1**			0.0
**Cancer origin**				**<0.0001**		
Non-small cell lung			6.45 [3.17; 13.15]		**1.86**	18.6
Colon			**1**			0
Stomach			2.84 [1.33; 6.08]		**1.04**	10.4
Ovary			1.09[0.51; 2.339]		**0.08**	0.8
Pancreas			4.55 [2.29; 9.02]		**1.51**	15.1
Cholangiocarcinoma			2.92 [0.72; 11.91]		**1.07**	10.7
Unknown			2.01 [0.16; 24.80]		**0.70**	7.0
Prostate			1.91 [0.92; 3.95]		**0.64**	6.4
Breast			1.82 [0.88; 3.75]		**0.60**	6.0
Bladder			3.98 [1.86; 8.50]		**1.38**	13.8
**Metastasis (M)**				**0.0007**		
No			**1**			0.0
Yes			2.41 [1.52; 3.81]		**0.88**	8.8
Missing and undetermined			2.31 [1.02; 5.22]		**0.84**	8.4
**Lymphocytes**				**0.03**		
<1500/mm3			1.56 [1.05; 2.33]		**0.44**	8.4
**Intercept**					**- 2.99**	

The prognostic score was calculated for 565 subjects and ranged from 0 to 63. In derivative population the parameters of the score were: mean 21.0, median 21.0, minimum 0 and maximum 51.7 in patients alive at one year and mean 34.4, median 32.6, minimum 0 and maximum 63.0 in those deceased. One-year observed mortality was 19.5% in subjects with a score of 21 and below, sensitivity was 84% and specificity 84%. One-year mortality rate was 70% when the score was 31 or more (sensitivity 60% and specificity 80%). Fig 2 shows the agreement between the predicted probability of death and the observed mortality rates on calibration plots.

### External validation of prognostic score

After exclusion of patients with lymphoma (110), those lost to follow-up (5) and those in whom the score could not be calculated (20), the validation cohort included 229 patients (Fig 1, Table 1). Malnutrition was found in 37 (16.2%) patients and nutritional risk in 127 (55.5%). After one year, 92 (40.2%) patients were deceased. The MNA with BMI as the 6^th^ question predicted mortality with an OR of 0.874 (95% CI 0.785–0.973, p = 0.01, AUC 0.595) for a one-point increase. The Full MNA had a better predictive value (OR for one-point increase 0.911, 95% CI 0.850–0.976, p = 0.008, AUC 0.606). The distribution parameters of the prognostic score were similar to those in the derivative population: mean 23.6, median 22.6, minimum 0 and maximum 51.5 in patients alive at one year and mean 32.7, median 32.1, minimum 0 and maximum 60.0 in those deceased.

The ROC curve of the score in the external cohort is presented in Fig 2. In the validation cohort, the AUC for the prognostic score was higher than that of the Full MNA (respectively, 0.698 and 0.606, p = 0.01), indicating a better prognostic performance.

## Discussion

In this model, we identified key elements associated with the risk of one-year mortality in older patients with an indication for chemotherapy based on food intake data, anthropometry, prescribed drug intake, lymphocyte count and cancer characteristics. The 1-year mortality prognostic score is simpler to assess than the MNA, includes information easily accessible at beside of the patient, and had better prognostic properties, as validated in an external cohort.

We have previously reviewed the role of malnutrition in worsening the vital prognosis of older patients with cancer, underlining the importance of cachexia [5]. Among the identified poor prognostic factors, weight loss, low leptin or low serum albumin and high C-reactive protein concentrations were frequently cited. Among multi-dimensional assessments of malnutrition or malnutrition risk, MNA or MNA items in older patients with diverse origin of cancer [2, 3, 10, 22] and PG-SGA in adult patients treated for gynecologic cancer [23] were predictors of short-term mortality. Several prognostic indexes with good performance have been developed in terminally ill subjects with cancer and contain nutritional indicators, mostly appetite assessment. However, the target populations of these studies had a very short-term mortality risk and chemotherapy treatments were stopped before the beginning of the follow-up. The Palliative Prognostic Score (PaP Score) was based on subjective clinical prediction of survival, Karnofsky Performance Status, anorexia, dyspnea, total white blood count and lymphocyte percentage [24]. The Terminal Cancer Prognostic score (TCP score), was constructed with three predictors: severe anorexia, severe diarrhea and mild confusion [25]. Another paper reported a prognostic score in subjects with a median survival lower than one month; the components were reduced oral intake, resting dyspnea, low performance status, leukocytosis, elevated bilirubin, creatinine and lactate dehydrogenase [26]. Quality of life was also associated to increased short-term mortality [27]. The Glasgow Prognostic score relies on inflammatory biological markers (C-reactive protein and serum Albumin) and predicts survival in advanced cancer [28]. In older patients this score was related to frailty assessed by the Edmonton frailty index [29]. The score developed in the present study was in line with the growing research on frailty assessment in older patient treated for cancer. Its originality was to predict not only short term but also mid-term mortality when chemotherapy treatment is decided.

The derivation and validation cohorts were considered as representative of the population of older patients undergoing chemotherapy treatment. However, subjects with the worst health status were probably not included since the oncologist may have already decided not to start chemotherapy. Indeed, the distribution of the MNA items suggested that the functional and mental status of these older patients were mainly preserved. The causes of deaths and the results of the prognostic model might have been very different if the cohort had included more bed-ridden or demented subjects. Thus the present model is not applicable to dependent patients. A limitation of this study was the absence of serum albumin in the candidate variables, although it has been shown to be an important prognostic marker in older age in community-living subjects [30, 31], in hospitalized older patients [32] and in other cachexia-inducing diseases [33, 34]. However, serum albumin determination prior to the decision to begin chemotherapy was not recommended within the timeframe of the study. From a pragmatic point of view, to be helpful to the clinician, potential predictors should be included in the usual set of clinical data available at the time of the decision.

The prognostic ranking of the tumor origins was similar to those reported in various national cancer mortality registers (National cancer institute (USA), http://www.cancer.gov/statistics, Institut National du Cancer, (France), http://www.e-cancer.fr/toutes-les-actualites/7324 and Cancer Research UK (UK), http://www.cancerresearchuk.org/cancer-info/cancerstats/survival/common-cancers/). Finally, while the tumor locations varied and the specificities of each were not detailed, the characteristics of dietary intake, including energy and protein intake, were not different across the tumor types in weight loss or in underweight patients [35], so the results of the present study are likely applicable in all older subjects receiving chemotherapy for cancer.

The influence of comorbidities, although considered as important in the frailty assessment, was not directly evaluated but can be approximated by using the number of prescribed drugs, which is a parameter that is very easy to obtain. Severe comorbidities according to the Charlson index have been found to be poor prognostic factors in colon cancer [36]. The mechanisms of this effect seem unclear since the causes of death were mainly the cancer itself. The effect is probably multifactorial owing to changes in the management of anticancer treatments due to the presence of comorbidity [37]. The number of disease-specific drugs used by older patients with depression was found to range from 1 to 3, from 1 to 4 in adults with diabetes mellitus and from 1 to 4 in those with hypertension [38]. Thus, the patients in this cohort who were taking one to two medications were likely to have no or only one comorbidity, and were very unlikely to have any severe comorbidity. This MNA item probably selects patients with no or mild comorbidity.

Like other disease associated cachexia [39] cancer cachexia is characterized by anorexia, early satiety, severe weight loss mainly at the expense of fat-free mass [40], weakness, anemia and edema. A correlation between the biochemical markers of cachexia and MNA score has been shown in patients with lung cancer [41]. Within the MNA items, signs associated with cancer cachexia were found to be mortality predictors in the present study. In a group of 170 weight-losing adults with advanced pancreatic cancers, individual features of cancer cachexia were found to be independent predictors of mortality. Meanwhile, weight loss was not an independent predictor of mortality [39]. Furthermore, weight loss in patients with cancer did not parallel the amount of their energy or protein intake [35]. Indeed, resting energy metabolism was significantly related to weight loss, while energy intake was not [35]. Calf circumference is a powerful prognostic factor of one-year mortality and has been shown to correlate with fat-free mass in older subjects and to predict sarcopenia [42]. It can always be measured and is probably a better marker of cachexia syndrome than weight loss.

In the score, reduced protein-rich food intake was found to contribute to the prediction of 1-year mortality, but reduced vegetable or liquid intake, even if associated with one-year mortality in univariate analysis, were not maintained in the final score. In a cluster study of dietary patterns retrieved in patients with cancer 6 to 8 months before death, intake of protein-rich foods such as meat was associated with less weight loss [43]. It is known that proteins provide greater satiety than other nutrients, especially fat. Thus, patients may specifically avoid protein-rich food because of severe cachexia and subsequent anorexia. This is the basis of corrective dietary counselling proposed for nutritional support in cachectic patients [6, 44].

As a conclusion, the key factors predictive of one-year mortality in this study included features of cancer cachexia, comorbidities, and the origin and advanced status of the tumor. These parameters are easy to retrieve, clinically relevant, and can be used by the clinician to make a prognosis before the onset of the chemotherapy.
